# Lupus Nephritis: Immune Cells and the Kidney Microenvironment

**DOI:** 10.34067/KID.0000000000000531

**Published:** 2024-08-09

**Authors:** Irene Chernova

**Affiliations:** Section of Nephrology, Department of Internal Medicine, Yale School of Medicine, New Haven, Connecticut

**Keywords:** glomerular endothelial cells, GN, hypoxia, lupus nephritis, mesangial cells, nephritis, osmolality, podocyte, renal tubular epithelial cells

## Abstract

Lupus nephritis (LN) is the most common major organ manifestation of the autoimmune disease SLE (lupus), with 10% of those afflicted progressing to ESKD. The kidney in LN is characterized by a significant immune infiltrate and proinflammatory cytokine milieu that affects intrinsic renal cells and is, in part, responsible for the tissue damage observed in LN. It is now increasingly appreciated that LN is not due to unidirectional immune cell activation with subsequent kidney damage. Rather, the kidney microenvironment influences the recruitment, survival, differentiation, and activation of immune cells, which, in turn, modify kidney cell function. This review covers how the biochemical environment of the kidney (*i.e*., low oxygen tension and hypertonicity) and unique kidney cell types affect the intrarenal immune cells in LN. The pathways used by intrinsic renal cells to interact with immune cells, such as antigen presentation and cytokine production, are discussed in detail. An understanding of these mechanisms can lead to the design of more kidney-targeted treatments and the avoidance of systemic immunosuppressive effects and may represent the next frontier of LN therapies.

## Introduction

Autoimmune diseases, such as SLE, are characterized by immune infiltration into various tissues, creating a scenario whereby immune cells must adapt to an unfamiliar environment. A tissue environment can be broken down into two factors: biochemical characteristics (*i.e*., low pH in the stomach, high oxygen tension in the lung) and a unique assortment of cell types. Infiltrating immune cells must successfully manage both these biochemical and cellular factors.

**Figure 1:**
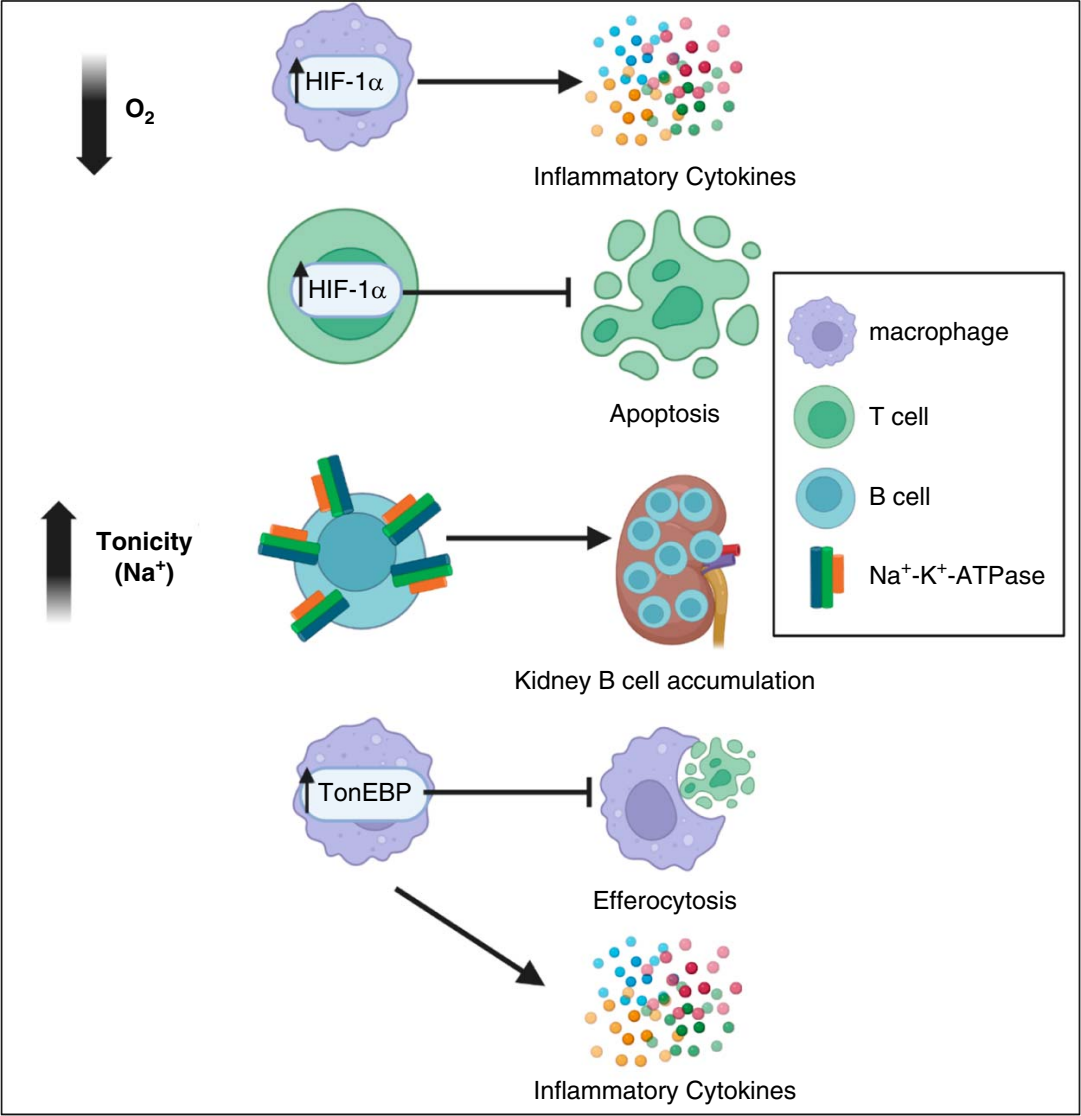
**Pathways upregulated by infiltrating immune cells to adapt to the biochemical environment of the lupus kidney.** In response to low oxygen tension, T cells and macrophages upregulate HIF-1*α*, resulting in decreased apoptosis and increased cytokine production, respectively. In response to hypertonicity, B cells upregulate Na^+^-K^+^-ATPase, resulting in improved survival in high-sodium conditions and B-cell accumulation in the LN kidney. Macrophages in LN upregulate TonEBP, a regulator of responses to hypertonic stress, leading to decreased efferocytosis (phagocytosis of apoptotic cells) and transcriptional upregulation of proinflammatory cytokines, although it is unknown whether hypertonicity is the trigger for increased TonEBP expression. HIF-1*α*, hypoxia-inducible factor 1*α*, LN, lupus nephritis; TonEBP, tonicity-responsive enhancer binding protein. Created with BioRender.com.

**Figure 2:**
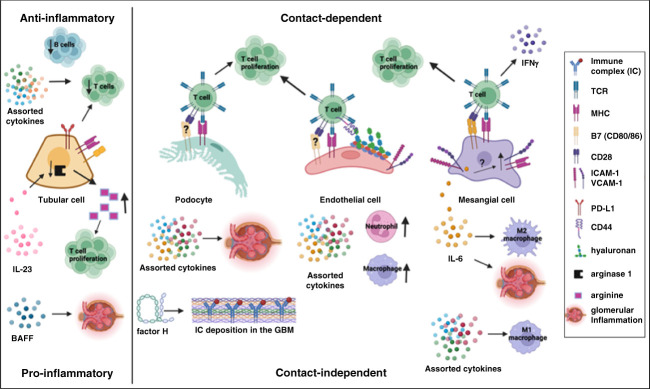
**Intrinsic renal cell interactions with infiltrating immune cells.** Selected contact-dependent and -independent effects of podocytes, endothelial cells, MCs, and RTECs on intrarenal immune components are summarized. Podocytes: cytokine secretion leads to glomerular inflammation, complement factor H secretion leads to IC deposition in GBM, and possible antigen presentation to T cells results in T-cell proliferation. GECs: cytokine secretion leads to neutrophil and macrophage recruitment; possible antigen presentation and interactions between endothelial glycocalyx hyaluronic acid with T-cell CD44 lead to enhanced T-cell proliferation/function. MCs: antigen presentation leads to T-cell proliferation and IFN-*γ* secretion. IL-6 production leads to glomerular inflammation, M1 macrophage polarization (in contrast to M2 macrophage polarization with other, undefined cytokines), and possibly MHC-II and ICAM-1 upregulation. RTECs: BAFF production leads to increased kidney inflammation, and reduced arginase 1 production in response to IL-23 in the lupus kidney leads to higher arginine levels and increased T-cell proliferation. RTECs express both stimulatory and inhibitory cell surface receptors, which, likely in combination with anti-inflammatory cytokines, lead to attenuated T- and B-cell proliferative responses. BAFF, B-cell activating factor; GBM, glomerular basement membrane; GEC, glomerular endothelial cell; IC, immune complex; ICAM-1, intracellular adhesion molecule 1; MC, mesangial cell; MHC, major histocompatibility complex; PDL-1, programmed death-ligand 1; RTEC, renal tubular epithelial cell; TCR, T-cell receptor; VCAM-1, vascular cell adhesion molecule 1. Created with BioRender.com.

Biochemically, the kidney presents a particularly hostile environment with hypertonicity and low oxygen tension not found elsewhere in the body.^1,2^ Recent work has identified immune cells' upregulation of molecules, such as tonicity-responsive enhancer binding protein (TonEBP), Na^+^-K^+^-ATPase, and hypoxia-inducible factor 1*α* (HIF-1*α*), to adapt to these stressors and will be covered in this review (Figure 1). On a cellular level, immune cells interact with native cells of the target organ in ways that are more complex than just inducing immune-mediated tissue damage. In fact, a large body of evidence demonstrates that intrinsic renal cells and immune cells signal to each other in contact-dependent and contact-independent ways, resulting in functional changes to both the resident renal cells and the infiltrating immune cells.^3–5^ How intrinsic renal cells influence the immune infiltrate is a particularly interesting part of this equation: If we can identify renal-specific pathways responsible for enhanced inflammation in lupus nephritis (LN), this opens the door for kidney-targeted treatments that are currently missing in this disease.^6^ The ability of glomerular and tubular cells to present antigen and make cytokines and survival factors that influence immune cell recruitment, proliferation, and function will be reviewed herein (Figure 2). Altogether this review will summarize how the unique biochemical and cellular kidney microenvironment shapes the renal immune infiltrate in LN and will highlight the opportunities for further research in this field.

## Biochemical Environment: Oxygen and Tonicity

The biochemical environment of the kidney presents a unique challenge for the infiltrating immune cells because they now need to survive in a hypoxic and hypertonic environment.^7,8^ In fact, LN kidneys present an even more hostile microenvironment than healthy kidneys because they have been shown to be comparatively more hypoxic, as determined by blood oxygenation level–dependent magnetic resonance imaging and immunostaining techniques.^2,9,10^ These findings are further supported by the increase in HIF-1*α* levels in the urine and HIF-1*α* expression in the glomerulus of patients with LN, which are positively correlated with histologic chronicity changes and renal pathology activity index, respectively.^11,12^ Both kidney resident cells, such as mesangial cells (MCs), and immune cells, such as macrophages and T cells, upregulate HIF-1*α* in LN.^2,12–14^ The expression in immune cells seems to be an adaptive survival mechanism, as HIF-1 blockade leads to a decrease in infiltrating immune cells and reverses kidney tissue injury.^2,14^ Intriguingly, the HIF-1 pathway protects against apoptosis during hypoxia in T cells while in macrophages, it seems to promote proinflammatory cytokine production.^2,14^ Immune cells' ability to coopt the HIF-1 pathway to survive in the LN kidney makes HIF-1 a compelling target for the treatment of this disease.

Another example of tissue-specific survival adaptation is the upregulation of Na^+^-K^+^-ATPase and its hypertonicity-sensitive *γ* subunit by intrarenal B cells in LN as a mechanism to survive the high-sodium kidney environment.^15^ Pharmacologic and genetic blockade of the ATPase results in a decrease in intrarenal B cells and amelioration of proteinuria, identifying Na^+^-K^+^-ATPase as an organ-specific therapeutic target.^15^ Kidney cells and infiltrating immune cells in LN also upregulate TonEBP (also known as the NF of activated T cells 5), a known regulator of cellular responses to hypertonic stress.^16^ Consistent with its role in mediating survival during hypertonic stress, TonEBP deficiency in myeloid cells leads to depletion of this cell lineage and ameliorates kidney injury in mouse SLE models.^16^ However, TonEBP is upregulated not just in the hypertonic medulla but also in the isotonic cortex of LN kidneys and has numerous other biological roles in leukocytes, so it is unclear whether its upregulation is directly related to how infiltrating immune cells survive hypertonicity in LN.^16^ Recent work in both mouse and human LN has shown that B versus T lymphocytes has differential localization between the cortex and medulla, with varying proximities to kidney intrinsic cell types.^15,17,18^ This localization clearly has implications for what survival pathways the cells would require, although these remain largely uncharacterized. Finally, high-salt diets are known to exacerbate SLE and LN in mouse models, at least, in part, by activating dendritic cells and inducing proinflammatory T-cell states.^19–21^ Whether the kidney interstitium promotes immune cell activation under these experimental conditions has not been examined. Additional research is required to understand how the kidney environment affects immune cell function in LN and what adaptation mechanisms immune cells use to survive and function in this unique environment.

## Podocytes

Podocytes, terminally differentiated epithelial cells of the glomerulus, are both damaged in some patients with LN (*i.e*., lupus podocytopathy) and increasingly recognized as contributors to LN pathogenesis.^22–24^ Podocytes are capable of producing multiple cytokines under basal conditions and evidence a unique pattern of proinflammatory chemokine and cytokine (*e.g*., IL-6, CXCL9, IFN-*γ*) secretion in the lupus kidney and when stimulated with inflammatory mediators found in the lupus kidney environment (*e.g*., IL-1*β*, CXCL13).^25–29^ Thus, podocytes seem to both contribute to and be influenced by the cytokine environment of the lupus kidney, although how exactly the multiple inputs and outputs are integrated *in vivo* remains unclear. For example, *in vitro* evidence demonstrates that podocyte-derived IL-6 negatively regulates neutrophil recruitment by effects on glomerular endothelial cells (GECs).^30^ By contrast, podocyte production of another cytokine, IL-23, has been correlated with increased inflammation in mouse models of nephritis.^31^ While many of the cytokines made by podocytes have known roles in immune cell recruitment and activation,^27,32–34^ it is difficult to definitively demonstrate that it is the podocyte-derived cytokine that is mediating a given immune function because there are multiple potential cellular sources of cytokines in the lupus kidney.^35^ It does seem that podocyte-specific calcium signaling can modulate immune complex deposition in LN, a key pathogenic step in the disease.^36^ Intriguingly, IgG antibody glycosylation can affect expression of the key molecule in the pathway, CAMK4, underscoring the bidirectional nature of immune:renal cell interactions.^37^ Finally, it is worth mentioning that podocytes are also capable of producing soluble, noncytokine immune molecules—the components of the complement cascade.^38^ Podocyte production of at least one complement component, complement factor H, has been suggested to play a role in immune complex deposition and LN pathogenesis in animal models, although definitive studies are pending.^39,40^

Podocytes are also believed to be nonhematopoietic antigen-presenting cells capable of directly stimulating renal-infiltrating T cells,^41,42^ although this view has been challenged more recently.^43,44^ The ability of podocytes to upregulate major histocompatibility complex (MHC)-I and MHC-II, molecules essential for antigen presentation to T cells, has been shown in a rat model of GN over 30 years ago,^41^ with more recent studies directly demonstrating that podocyte-restricted MHC and antigen presentation can activate T cells *in vitro* and *in vivo*.^42,45,46^ Moreover, T-cell costimulatory and inhibitory molecules of the B7 family (CD80/CD86, PD-L1, PD-L2) are known to be expressed in podocytes, with CD80/86 expression seen in mouse and human LN kidneys and correlating with severity of proteinuria in human LN.^47–50^ Intriguingly, the effect of podocyte B7 on proteinuria was independent of the adaptive immune system because severe combined immunodeficiency disease mice lacking T cells and B cells still evidenced podocyte B7 upregulation upon LPS treatment.^48^ Recently, several careful validation studies have questioned B7 expression on podocytes in both mouse and human LN and concluded that these cells do not express B7 after all.^43,44^ Clinical trials targeting the B7 pathway with the drug abatacept also failed to show efficacy in LN, and any renoprotective effects in such trials may be explained by inhibiting T-cell activation independent of podocytes.^51,52^ Because the signals required by kidney-infiltrating T cells in LN are not fully known,^53^ it is unclear whether lack of B7 on podocytes would mean that they cannot serve as antigen-presenting cells in the glomerulus. Further studies are needed to clarify this important point.

## GECs

Endothelial cells are a specialized single cell layer lining vessels across the body; in the glomerulus, they form the filtration barrier along with the glomerular basement membrane and podocytes.^54,55^ When treated with cytokines known to be involved in LN pathogenesis or sera from patients with lupus, GECs significantly increase production of multiple proinflammatory cytokines (*e.g*., IL-6, TNF-*α*, IFN-*β*) and leukocyte adhesion molecules (intracellular adhesion molecule 1 [ICAM-1], vascular cell adhesion molecule 1).^56–58^ Moreover, some of the conditions resulted in increased neutrophil chemotaxis and adhesion to GECs, suggesting that proinflammatory GEC activation may play a role in the recruitment of this cell type.^56,57^ Other authors have demonstrated the ability of GECs to produce cytokines, such as CX3CL1, that are known potent leukocyte chemoattractants,^58–60^ with recent work showing that this may lead to macrophage infiltration of glomeruli in an antibody-induced LN model.^61^ Kadoya *et al.* defined a novel role for endothelial glycocalyx, the membrane-bound layer of glycoproteins on endothelial cells, in the pathogenesis of LN.^62^ The glycocalyx in lupus-prone mice was substantially thicker than in healthy animals and capable of recruiting activated memory T cells, which bound their receptor CD44 to the hyaluronic acid component of the glycocalyx.^62^ Inhibition of hyaluronic acid *in vivo* led to decreased infiltration by CD3^+^CD4^−^CD8^−^ T cells and reduced tissue damage, highlighting the potential of tissue-directed approaches in the treatment of LN.^63^ In parallel to podocytes, endothelial cells are also capable of expressing MHC-I, MHC-II, and the costimulatory molecules CD80/CD86 and capable of stimulating T cells,^64–68^ although some older reports have questioned whether endothelial cells are able to express CD80/CD86.^69,70^ Just as for podocytes, the antigen-presenting function of GECs remains to be fully elucidated.

## MCs

MCs comprise roughly one third of all cells in the glomerulus, occupying the space between glomerular arterioles and the glomerular basement membrane and providing structural support by mesangial matrix secretion.^71–73^ Like podocytes and endothelial cells, MCs are capable of upregulating MHC, CD80, CD40, and adhesion molecules ICAM-1 and vascular cell adhesion molecule 1 when stimulated with proinflammatory cytokines, such as IFN-*γ*, that is commonly found in the LN kidney.^74–78^ These conditions can lead to T-cell proliferation and differentiation into an IFN-*γ*–producing T-cell subset, presumably further perpetuating this MC–T-cell activation cycle.^75^ Cytokine-stimulated MCs can also influence macrophage polarization, with the proinflammatory M1 macrophage phenotype being driven by different inputs than those leading to the anti-inflammatory M2 phenotype.^79^

Like GECs and podocytes, MCs have been reported to make a variety of cytokines, although most of the literature highlights their production of one proinflammatory cytokine: IL-6.^79–88^ In fact, studies using more modern confocal microscopy techniques suggest that MCs are the primary producers of IL-6 in the lupus kidney, with production limited to animals with severe proteinuric LN.^81^ Intriguingly, even other cytokines made by MCs, such as IFN-*α* and IFN-*β*, may serve primarily to enhance the cells' production of IL-6 in an autocrine proinflammatory loop.^82^ Evidence also shows that anti-dsDNA autoantibody binding to MCs serves as a signal for IL-6 upregulation.^89^ IL-6 seems to have a role in glomerular leukocyte infiltration and exogenous IL-6 has been reported to upregulate MHC-II and ICAM-1, raising the intriguing possibility that cytokine-producing and antigen-presenting roles of glomerular cells may be inter-related.^90,91^ Other cytokines made by MCs include CXCL1 (neutrophil chemoattractant), CCL2 (monocyte chemoattractant), granulocyte-macrophage colony-stimulating factor (promotes myeloid cell maturation), and B-cell survival factors B-cell activating factor (BAFF) and a proliferation-inducing ligand.^79,88,92–96^

It is worth ending on a word of caution regarding cytokine profiles in intrinsic glomerular cells, in LN and in other kidney diseases. First, many older publications are not able to definitively distinguish glomerular cell type given the techniques of the time, making data from those sources inherently limited. Second, Sung *et al.* showed that cytokine production by glomerular cells *in situ* may differ from the cytokines made by cells from isolated, decapsulated glomeruli.^81^ Newer techniques, such as spatial RNA sequencing and multicolor confocal microscopy approaches, are rapidly enhancing our knowledge in ways that account for the 3D structure and cell:cell interactions in the glomerulus, although more work is needed to capture the full spectrum of kidney inflammatory mediators in this heterogeneous disorder.^81^

## Renal Tubular Epithelial Cells

Tubulointerstitial inflammation independently correlates with renal survival in LN, raising questions about the immunological roles of renal tubular epithelial cells (RTECs), the predominant cell type of the tubulointerstitium.^97,98^ Immunological roles of RTECs have been recently appreciated, with the cells appearing to play both pro- and anti-inflammatory parts. Proximal tubular epithelial cells upregulate molecules implicated in antigen presentation and are capable of stimulating CD4^+^ T cells *in vitro*.^99^ RTECs appear to be the main producers of IFN-*α* and are also responsive to this cytokine, potentially indicating the presence of an autocrine loop.^100^ IFN-*α* signaling can lead to upregulation of molecules, such as MHC, once again highlighting cytokines' putative role in facilitating antigen presentation.^100^ An IFN-response signature in tubular cells (and in keratinocytes) can successfully differentiate patients with LN from healthy controls and predict response to treatment.^101^ RTECs are also capable of making a profile of inflammatory cytokines that is similar to that made by intrinsic cells of the glomerulus (*i.e*., IL-6, CX3CL1, TNF-*α*, IL-1*β*) and that can drive chemotaxis of certain target leukocytes, such as dendritic cells.^102–105^ Intriguingly, conditioned media from MCs could enhance this cytokine production by RTECs, showcasing the likely complexity of proinflammatory cell:cell networks in the LN kidney.^102^ RTECs cultured under hypoxic conditions activate dendritic cells, resulting in increased production of inflammatory cytokines IL-1*β* and IL-18.^106^ Although this pathway has not been studied directly in LN, it may be worth investigating given that LN kidneys are more hypoxic than healthy controls, as discussed above.^2,9^ RTECs may also play a role in CD4^+^ T-cell recruitment by secretion of CXCL9 and CXCL10 because steroid blockade of this pathway could ameliorate disease in a non-lupus GN model.^107^ Finally, RTECs can make BAFF, the expression of which correlates with LN activity.^108^ This is potentially a particularly important finding given the use of BAFF blockade clinically in lupus and the general paucity of data on kidney cell:B-cell interactions despite the known contribution of B cells to LN pathology.^109^

Unlike for intrinsic cells of the glomerulus, there is a body of evidence that RTECs can attenuate immune responses. While there is some evidence that RTEs can express MHC and costimulatory molecules that help drive T-cell activation, other studies show expression of inhibitory receptors.^110–114^ In these studies, RTECs inhibit proliferative T- and B-cell responses and skew lymphocyte cytokine profiles, perhaps through cell–cell contacts.^112–115^ Human activated proximal tubular epithelial cells also inhibit the differentiation of monocytes and lead to higher levels of secretion of IL-10, an anti-inflammatory cytokine.^116^ Finally, RTECs seem to have a metabolic immunosuppressive role that is negated in the lupus proinflammatory environment. RTECs make arginase 1, thereby lowering the amount of arginine in the environment and limiting the use of this nutrient by infiltrating T cells.^117^ In the lupus kidney environment, high IL-23 levels suppress arginase 1 production by RTECs, resulting in more arginine availability and increased local T-cell proliferation and activation.^117^ In sum, however, caution is warranted as many of the described pathways have not been investigated in a lupus model, and thus the balance of pro- and anti-inflammatory functions of RTECs in lupus remains unknown.

## Conclusions

The kidney is a unique, hostile tissue microenvironment that hosts few immune cells under conditions of homeostasis. The infiltrating immune cells in LN must adapt to the biochemical environment of the kidney while simultaneously receiving both contact-dependent and soluble signals supporting their function. The intrinsic cells of the kidney can provide these signals to the immune cells while in some cases also dampening such immune responses. The temporal nature and directionality of these signals remain incompletely characterized, although it is clear that there are cytokine-mediated networks between kidney intrinsic cell types and bidirectional communication between intrinsic renal and infiltrating immune cells. Further mechanistic research is needed to define these renal:immune networks to enable us to target intrarenal inflammation without affecting systemic immunity,^118^ giving us new powerful weapons in the fight against LN.
